# Suppressed Growth of (Fe, Cr_,_ Co_,_ Ni_,_ Cu)Sn_2_ Intermetallic Compound at Interface between Sn-3.0Ag-0.5Cu Solder and FeCoNiCrCu_0.5_ Substrate during Solid-state Aging

**DOI:** 10.1038/s41598-019-46757-w

**Published:** 2019-07-15

**Authors:** Yu-An Shen, Chun-Ming Lin, Jiahui Li, Runhua Gao, Hiroshi Nishikawa

**Affiliations:** 1 Joining and Welding Research Institute (JWRI), Osaka University, Osaka, 5600047 Japan; 2 Department of Mechanical Engineering, Minghsin University of Science and Technology, Hsinchu, 30401 Taiwan Republic of China; 3 Department of Aviation Mechanical Engineering, China University of Science and Technology, Hsinchu, 312 Taiwan Republic of China; 4 Graduate School of Engineering, Osaka University, Osaka, Japan

**Keywords:** Electronic devices, Metals and alloys, Mechanical engineering

## Abstract

High-entropy alloys (HEAs) are promising materials for next-generation applications because of their mechanical properties, excellent high-temperature stability, and resistance against oxidation and corrosion. Although many researchers have investigated high-temperature HEA applications, few have considered low-temperature applications. Here we demonstrate an unprecedented intermetallic compound of (Fe, Cr, Co, Ni, Cu)Sn_2_ at the interface between Sn-3.0Ag-0.5Cu (SAC) solder and FeCoNiCrCu_0.5_ HEA substrate after reflow at 400 °C. Significantly suppressed growth of intermetallic compound without detachment from the substrate was observed during thermal aging at 150 °C for 150 h. Sn grains with an average grain size of at least 380 μm are observed. The results reveal a completely new application for the fields of Sn-Ag-Cu solder and HEA materials.

## Introduction

Various studies indicate that high-entropy alloy (HEA), which is composed of at least five principal elements, has excellent mechanical properties and offers the advantages of high-temperature stability and resistance to oxidation and corrosion^1–5^. A recent study of FeCoNiCrCu_0.5_, a face-centered cubic (FCC) structure with a segregation phase of Cu, showed that the hardness of this structure remained unchanged after various heat treatments, and that its corrosion properties in 3.5% NaCl were better than those of AISI 304L stainless steel^2,6^. Although high-temperature applications have been studied extensively, research into utilization in low-temperature industries is rare.

A common solder for electronics packaging industry is the Sn-Ag-Cu (SAC) solder ball^7–9^, properties of which include a low melting point (217–220 °C), properly thermal behavior, shear strength, and wetting on Cu or Ni substrates. However, fracture on the brittle intermetallic compound (IMC) layer, with a high growth rate during solid-state aging tests, severely impacts the mechanical reliability of joints formed with SAC solder^7,10^. A FeCoNiCrCu_0.5_ HEA has limited atoms available for Sn-based IMC formation, and is thus a promising solution to the problem of high growth rate. In addition, if a SAC alloy could be soldered on to a FeCoNiCrCu_0.5_ HEA, a novel system offering the simultaneous advantages of SAC and HEA substrates could be developed, facilitating new applications in industry.

Herein, we investigate a Sn-3.0Ag-0.5Cu-FeCoNiCrCu_0.5_ HEA substrate (SAC-HEA) structure using a 400 °C reflow process. The growth rate of the IMC at the interface is examined by an aging test conducted at 150 °C for 150 h (approximately 1 week). SAC solder on Cu substrates (SAC-Cu) serves as a benchmark. In our experiment, a SAC solder ball 0.76 μm in diameter is successfully soldered onto a FeCoNiCrCu_0.5_ HEA substrate using a 400 °C reflow profile in a reflow furnace (Fig. S1). Reflow is a process used to melt a solder ball, to achieve solder-on-substrate joining. The HEA substrate is from a HEA bulk fabricated by melting suitable amounts of the pure (99.99%) elements in an argon atmosphere in an arc furnace. The bulk is cut into HEA substrates of 3 mm × 6 mm × 3 mm. Samples are packaged in Aluminum foil and put into an oil bath at 150 °C for an aging test of 150 h. The experimental method and materials used are described in detail in the Method section.

**Figure 1:**
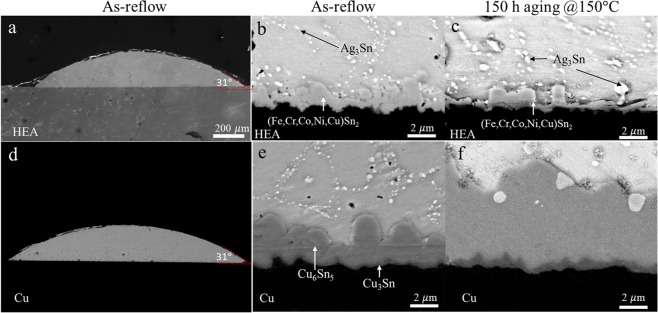
Cross-sectional images of each of the samples. (**a**) A SEM image of Sn-3.0Ag-0.5Cu solder with a contact angle of 31° on a HEA substrate. (**b**) A cross-sectional image of back-scattered electrons shows that the intermetallic compound at the interface is (Fe_0.5_Cr_0.22_Co_0.19_Ni_0.06_Cu_0.03_)Sn_2_ and the small white grains are Ag_3_Sn in SAC-HEA. (**c**) A cross-sectional image of a SAC-HEA sample after 150 hr aging at 150 °C. (**d**) A SEM image of SAC with a contact angle of 31° on a Cu substrate. (**e**) A cross-sectional image of back scattered electron shows that the intermetallic compound at the interface is Cu_3_Sn and Cu_6_Sn_5_ on a Cu substrate. (**f**) A cross-sectional image of a SAC-Cu sample after 150 hr aging at 150 °C.

**Figure 2:**
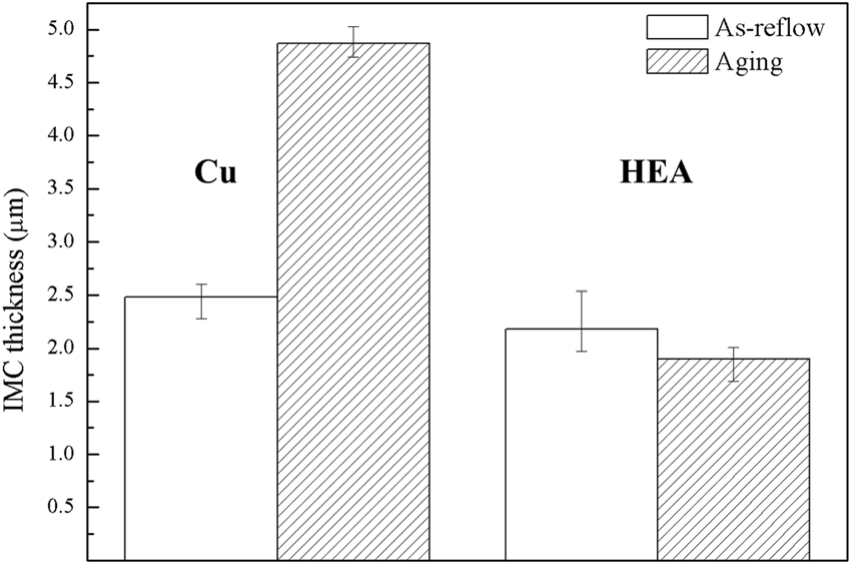
IMC average thickness before and after 150 hr aging at 150 °C in each sample. The IMC thickness of SAC-Cu is 2.48 μm and 4.67 μm before and after aging, respectively. The IMC thickness of SAC-HEA is 2.18 μm and 1.9 μm before and after aging, respectively.

In this study, we focus on the IMC behavior at the interfaces between the SAC solder and the substrates. Using a scanning electron microscope (SEM, JOEL 7800, Japan) to observe the cross-sectional back-scattered images (BEIs), we find that although the reflow temperature was 400 °C, the thickness of IMC of (Fe, Cr_,_ Co_,_ Ni_,_ Cu)Sn_2_, which is identified by energy dispersive X-ray spectrometer (EDS) and electron probe microanalyzer (EPMA, JOEL JXA-8530F, Japan), in SAC-HEA is approximately equal to the thickness of Cu-Sn IMC in SAC-Cu (Fig. 1b,e). Additionally, the contact angle of SAC-HEA is 31°, equal to that of SAC-Cu, as shown in Fig. 1a,d. FeSn_2_ IMC was formed at the Sn-Fe interface after 600 s at 400 °C^11^; therefore, Fe-Sn based IMC was formed at the SAC-HEA interface after reflow for 2 min at 400 °C. The main matrix phase of FeCoNiCrCu_0.5_ HEA had FCC structure with 24% Fe, 24% Co, 25% Ni, 22% Cr and 9% Cu (in at. %). Hence, the Fe atoms are replaced with Co, Ni, Cr, and Cu atoms in FeSn_2_ IMC, forming (Fe, Cr_,_ Co_,_ Ni_,_ Cu)Sn_2_ at the SAC-HEA interface. The measurement of the elements in the IMC layers is summarized in Table 1. Additionally, after reflow at 250 °C for 2 min, Fig. S2 shows the IMC at the SAC-HEA interface is (Cu,Ni)_6_Sn_5_^12^, rather than (Fe, Cr, Co, Ni, Cu)Sn_2_. This provides the evidence that 400 °C reflow is the key to (Fe, Cr_,_ Co_,_ Ni_,_ Cu)Sn_2_ formation. Figure 1c,f show the BEIs of SAC-HEA and SAC-Cu, respectively, after 150 h aging at 150 °C. It can be seen that the IMC at the SAC-HEA interface grew rarely, but that at the SAC-Cu interface did thicken considerably. In Fig. 2, the IMC thickness at the SAC-Cu interface changes from 2.48 to 4.67 μm, but that at the SAC-HEA interface does not change significantly (from 2.18 to 1.9 μm). In this study, there are six samples for both as-reflow and aging conditions. Three random areas are selected on each sample to acquire the average thickness of IMC. IMC thickness is lower after thermal aging caused by a margin of error during calculation rather than an actual reduction in the IMC thickness. Thus the difference of IMC thickness in as-reflow and aging samples can be ignored. The rapid growth of Sn-Cu IMC is commonly observed at 150 °C aging in SAC-Cu samples, whereas, (Fe, Cr, Co, Ni, Cu)Sn_2_ is not formed at 250 °C reflow, let alone by 150 °C aging in SAC-HEA samples. In other words, if the IMC of (Fe, Cr, Co, Ni, Cu)Sn_2_ did not form at 250 °C, the growth should be very limited during the aging process at 150 °C. Thus, the IMC formation at the SAC-HEA interface is suppressed remarkably during thermal aging process.

**Table 1:** The elements in (Fe, Cr, Co, Ni, Cu)Sn_2_ IMC (At. %) before and after thermal aging.

Elements	Fe	Cr	Co	Ni	Cu	Sn	Conditions
At. %	18.58	7.51	5.06	1.90	1.24	65.71	As-reflow
	17.1	6.27	5.44	1.85	1.21	68.13	Aging

(Fe, Cr, Co, Ni, Cu): Sn = 1: 1.92 for as-reflow.

(Fe, Cr, Co, Ni, Cu): Sn = 1: 2.13 for aging.

**Figure 3:**
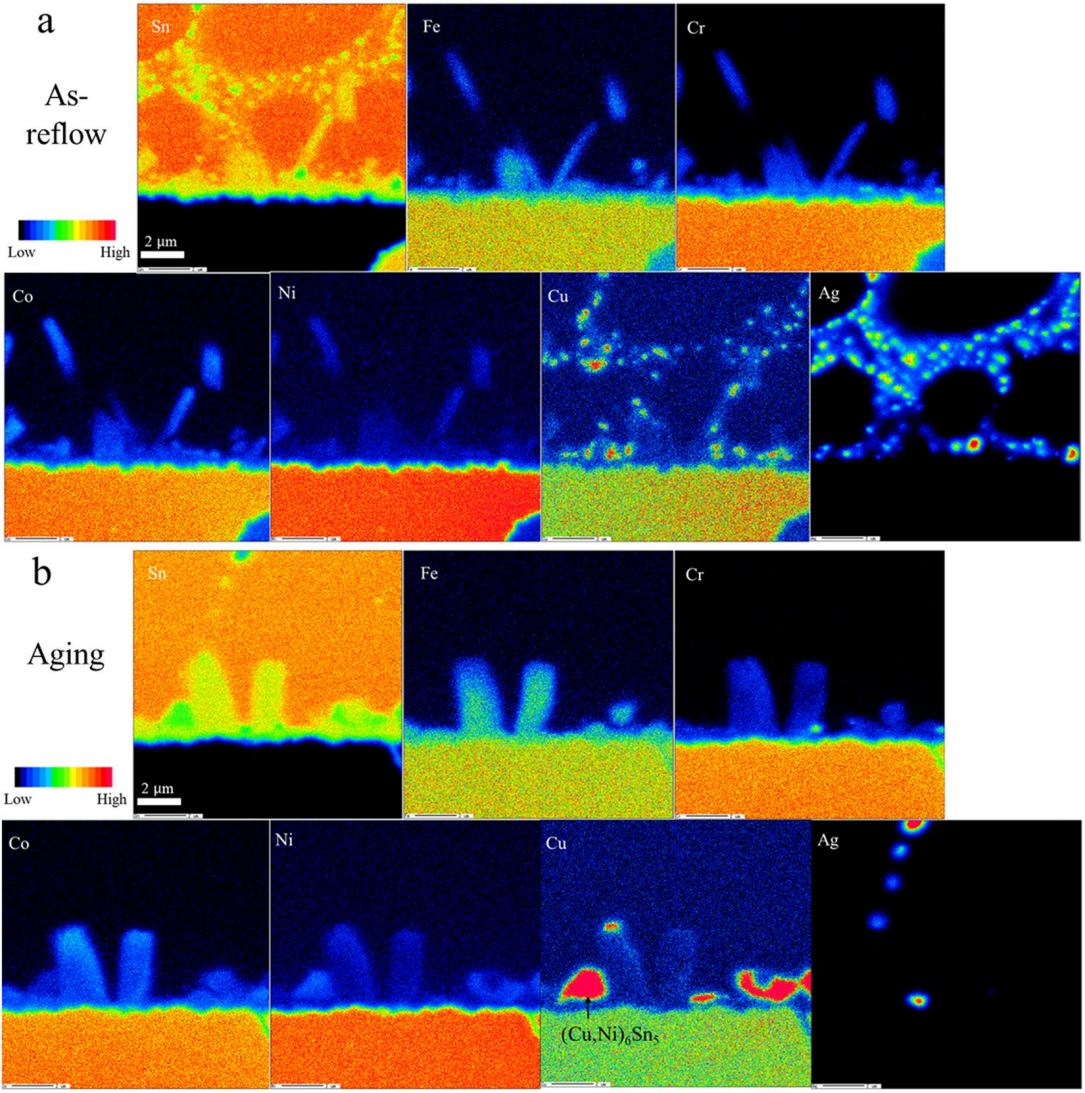
Distribution of elements before and after aging in SAC-HEA samples. (**a**) Images of electron probe microanalyzer for the distribution of each element in a SAC-HEA sample before aging and (**b**) after aging. Color bar shows the low at% to high at%.

The distribution of elements in SAC-HEA before and after 150 h aging at 150 °C is analyzed by EPMA mapping, as shown in Fig. 3a,b, respectively. In Fig. 3a, we can observe that Sn, Fe, Co, Ni, Cr, and Cu compose the IMC at the interface; some Ag atoms react with Sn to compose Ag_3_Sn IMC, whereas the other Ag atoms separate around the grains of Ag_3_Sn IMC in the SAC solder. Moreover, Cu atoms randomly separate in SAC solder. After 150 h aging at 150 °C, Fig. 3b shows that the Sn-HEA interface is still comprised of (Fe, Co, Ni, Cr, Cu)Sn_2_ IMC. However, (Cu,Ni)_6_Sn_5_ IMC grains are detected upon the (Fe, Co, Ni, Cr, Cu)Sn_2_ IMC during the aging process. Interestingly, the Ag separation near Ag_3_Sn disappears. The Ag solubility in Sn at room temperature is 0.052 wt.%, causing Ag atoms to precipitate out in the SAC solder with 3.0 wt.% Ag. While the grain coarsening of Ag_3_Sn occurs during the aging at 150 °C, the separating Ag atoms became the source for Ag_3_Sn growth, leading to the larger Ag_3_Sn grains observed in Figs 1c and 3b.

**Figure 4:**
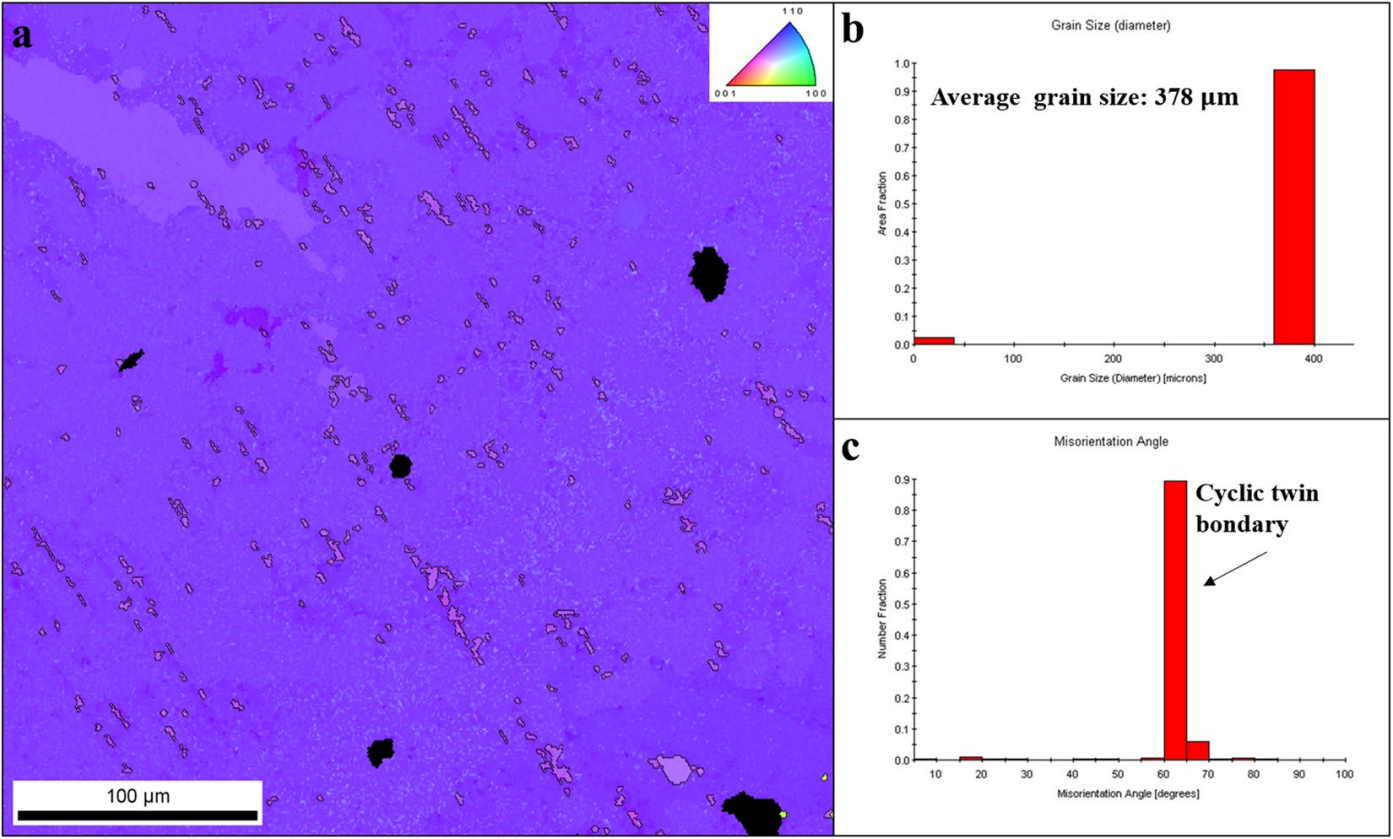
EBSD analysis for Sn grain orientation and size in SAC-HEA. (**a**) An orientation image map by Sn inversed pole figure in the normal direction. (**b**) The distribution of grain size shows that the average grain size is relative, at least 380 μm. (**c**) The distribution of misorientation of grain boundaries shows that most grain boundaries are of the cyclic twin boundary.

A Sn orientation image mapping (OIM) by electron backscattered diffraction (EBSD, TSL and OIM Analysis, Japan) demonstrates a large grain in a SAC-HEA sample (Fig. 4a). Figure 4b shows that the average grain size is at least 378 μm in the OIM of Fig. 4a. Grain boundary is critical to the reliability properties of solder joints. Tasooji, *et al*. clearly showed that, because the diffusivity of Cu through the Sn grain boundary is much higher than that through the Sn lattice, significant atomic diffusion occurs through high-angle grain boundary during electromigration, causing considerable IMC formation in the solder and exhausting the substrate^13^. Conversely, Sn has an anisotropy coefficient of thermal expansion (CTE) caused by its body-center-tetragonal structure. The CTE of [001] is approximately 15 times larger than that of [100]^14^. The CTE mismatch between grains causes significant crack propagation along the grain boundaries in the Sn-rich solder^15^. Moreover, Fig. 4c shows the misorientation of grain boundaries in the OIM of Fig. 4a. The grain boundaries are of the cyclic twin boundary (CTB) type with the coherent boundary structure commonly exhibited in Sn-rich solders, but not commonly seen in Sn-Cu solders^16,17^. Shen, *et al*. demonstrated that atoms hardly diffuse along CTB due to current stressing, i.e., electromigration^18^. Hence, the distribution of large Sn grains is highly beneficial to the mechanical and electromigration reliability of SAC-HEA. Although there are few Sn grain boundaries in SAC-HEA, those that are present are mostly CTB which could prevent SAC solder from experiencing crack propagation and electromigration.

## Conclusion

These findings not only provide a method to fabricate SAC-HEA, but also shed light on the reactions of SAC solder with HEA and the Sn microstructures in the SAC solder. The IMC formation of (Fe, Cr_,_ Co_,_ Ni_,_ Cu)Sn_2_ at the interface is key to the SAC-HEA samples, and its excellent stability suppressed IMC growth at 150 °C. Moreover, the average grain size is approximately 380 μm and CTBs are found in the Sn solder on the HEA substrate. The results in this study are unprecedented in the HEA and solder joint fields.

## Method

### Materials

The ingot for FeCoNiCrCu_0.5_ HEAS was melted in an argon atmosphere in an arc furnace with a mixture of appropriate amounts of high-purity elements (99.99%). The ingots were obtained in a copper mold. Each sample was reversed and re-melted four times to assure chemical homogeneity. The final samples were button-shaped, approximately 8 mm thick, with a shiny surface. The microstructure and chemical composition of the alloys were analyzed by scanning electron microscope (SEM, JEOL JSM-5410) and energy dispersive spectrometer (EDS). Commercially fabricated Cu substrates 16 mm × 16 mm × 0.5 mm in dimension, and ball-shaped Sn-3.0Ag-0.5Cu solders with a diameter of 0.76 mm, were used.

### Thickness of Intermetallic compounds

We used software to measure the areas of interface between Sn-3.0Ag-0.5Cu solder balls and the substrates. Three different 18-μm-wide regions were measured in each sample. Then, the areas are divided by the width (18 μm) to calculate the thicknesses. There are three samples for SAC-HEA and SAC-Cu, respectively. The equation can be expressed as follows:

$$T=\frac{A}{18\,\mu m},$$

where T is the IMC thickness, A is the measured area of IMC at the interface, and 18 μm is the measured width.

## Supplementary information

Suppressed Growth of (Fe, Cr, Co, Ni, Cu)Sn2 Intermetallic Compound at Interface between Sn-3.0Ag-0.5Cu Solder and FeCoNiCrCu0.5 Substrate during Solid-state Aging

## Data Availability

The data that support the findings of this study are available from the corresponding authors upon reasonable request.

## References

[1] Yeh J-W (2004). Nanostructured High-Entropy Alloys with Multiple Principal Elements: Novel Alloy Design Concepts and Outcomes. Adv. Eng. Mater..

[2] Hsu Y-J, Chiang W-C, Wu J-K (2005). Corrosion behavior of FeCoNiCrCux high-entropy alloys in 3.5% sodium chloride solution. Mater. Chem. Phys..

[3] Li Z, Pradeep KG, Deng Y, Raabe D, Tasan CC (2016). Metastable high-entropy dual-phase alloys overcome the strength–ductility trade-off. Nature.

[4] Cheng K-C, Chen J-H, Stadler S, Chen S-H (2019). Properties of atomized AlCoCrFeNi high-entropy alloy powders and their phase-adjustable coatings prepared via plasma spray process. Appl. Surf. Sci..

[5] Ye YF, Wang Q, Lu J, Liu CT, Yang Y (2016). High-entropy alloy: challenges and prospects. Mater. Today.

[6] Lin C-M, Tsai H-L (2010). Equilibrium phase of high-entropy FeCoNiCrCu0.5 alloy at elevated temperature. J. Alloys Compd..

[7] Kim KS, Huh SH, Suganuma K (2003). Effects of intermetallic compounds on properties of Sn–Ag–Cu lead-free soldered joints. J. Alloys Compd..

[8] Zhang L, Wang ZG, Shang JK (2007). Current-induced weakening of Sn3.5Ag0.7Cu Pb-free solder joints. Scr. Mater..

[9] Shen Y-A, Ouyang F-Y, Chen C (2019). Effect of Sn grain orientation on growth of Cu-Sn intermetallic compounds during thermomigration in Cu-Sn2.3Ag-Ni microbumps. Mater. Lett..

[10] Zeng K, Tu KN (2002). Six cases of reliability study of Pb-free solder joints in electronic packaging technology. Mater. Sci. Eng. R Reports.

[11] Protsenko P, Terlain A, Traskine V, Eustathopoulos N (2001). The role of intermetallics in wetting in metallic systems. Scr. Mater..

[12] Shen Y-A, Lin C-M, Li J, He S, Nishikawa H (2019). Effect of FeCoNiCrCu0.5 High-entropy-alloy Substrate on Sn Grain Size in Sn-3.0Ag-0.5Cu Solder. Sci. Rep..

[13] Tasooji A, Lara L, Lee K (2014). Effect of Grain Boundary Misorientation on Electromigration in Lead-Free Solder Joints. J. Electron. Mater..

[14] Bieler TR (2008). Influence of Sn Grain Size and Orientation on the Thermomechanical Response and Reliability of Pb-free Solder Joints. IEEE Trans. Components Packag. Technol..

[15] Shnawah DA, Sabri MFM, Badruddin IA (2012). A review on thermal cycling and drop impact reliability of SAC solder joint in portable electronic products. Microelectron. Reliab..

[16] Lehman LP, Xing Y, Bieler TR, Cotts EJ (2010). Cyclic twin nucleation in tin-based solder alloys. Acta Mater..

[17] Shen Y-A, Zhou S, Nishikawa H (2019). Preferred orientation of Bi and effect of Sn-Bi microstructure on mechanical and thermomechanical properties in eutectic Sn-Bi alloy. Materialia.

[18] Shen Y-A, Chen C (2017). Effect of Sn grain orientation on formation of Cu6Sn5 intermetallic compounds during electromigration. Scr. Mater..

