# Supplementary material for: Suppressed Growth of (Fe, Cr, Co, Ni, Cu)Sn2 Intermetallic Compound at Interface between Sn-3.0Ag-0.5Cu Solder and FeCoNiCrCu0.5 Substrate during Solid-state Aging
Source: Sci Rep. 2019 Jul 15;9:10210. doi: 10.1038/s41598-019-46757-w (PMC6629638; doi:10.1038/s41598-019-46757-w)
Supplement: Supplementary file 1 — Suppressed Growth of (Fe, Cr, Co, Ni, Cu)Sn2 Intermetallic Compound at Interface between Sn-3.0Ag-0.5Cu Solder and FeCoNiCrCu0.5 Substrate during Solid-state Aging [file 41598_2019_46757_MOESM1_ESM.docx]

## Supplementary information

**Suppressed Growth of (Fe, Cr_,_ Co_,_ Ni_,_ Cu)Sn_2_ Intermetallic Compound at Interface between Sn-3.0Ag-0.5Cu Solder and FeCoNiCrCu_0.5_ Substrate during Solid-state Aging**

Yu-An Shen^1*^, Chun-Ming Lin^2,3^, Jiahui Li^1^, Runhua Gao ^4^, Hiroshi Nishikawa^1^

1 Jointing and Welding Research Institute (JWRI), Osaka University, Osaka, 5600047, Japan

2 School of Mechanical Engineering, Hefei University of Technology, Hefei, 230009, China

3 Department of Aviation Mechanical Engineering, China University of Science and Technology, Hsinchu, 312, Taiwan

4 Graduate School of Engineering, Osaka University, Osaka, Japan

* Corresponding author. Tel.: +81 668798685;

E-mail address: [yashen@jwri.osaka-u.ac.jp](mailto:yashen@jwri.osaka-u.ac.jp) (Y.A. Shen).

***1. Supplementary Figures***

**

**

**Supplementary Fig. S1︱ Reflow profile.** The black line is the reflow profile of 400$^{\circ}$C for fabricating SAC-HEA samples. The red line is the reflow profile of 250$^{\circ}$C for fabricating SAC-Cu samples. Additionally, The SAC-HEA sample in Figure S2 is fabricated by the reflow profile of 250$^{\circ}$C. The heating rate is 2$^{\circ}$C/s, the peak time is 2 mins, and the cooling rate is 0.5$^{\circ}$C.


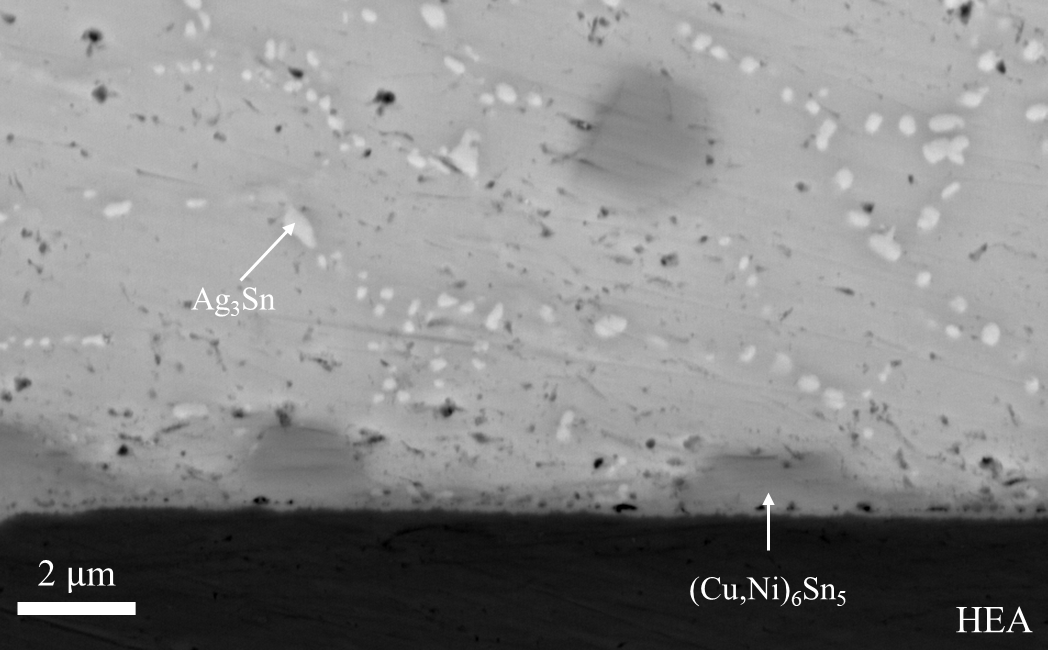


**Supplementary Fig. S2︱** **BEI of SAC-HEA fabricated by 250**$\boldsymbol{^{\circ}}$**C reflow.** The IMC at the interface is (Cu,Ni)_6_Sn_5_ which is identified by EDS.
